# Rikkunshito ameliorates colonic dysfunction and hormonal imbalance in an IBS mouse model with modulation of transient receptor potential channel activity

**DOI:** 10.3389/fphar.2026.1813619

**Published:** 2026-05-22

**Authors:** Woo-Gyun Choi, Na Ri Choi, Daehwa Jung, Sang Chan Kim, Joo Han Woo, Byung Joo Kim

**Affiliations:** 1 Department of Longevity and Biofunctional Medicine, Pusan National University School of Korean Medicine, Yangsan, Republic of Korea; 2 Department of Pharmaceutical Engineering, Daegu Hanny University, Gyeongsan, Republic of Korea; 3 College of Oriental Medicine, Daegu Hanny University, Gyeongsan, Republic of Korea; 4 Department of Physiology, Dongguk University College of Medicine, Gyeongju, Republic of Korea

**Keywords:** colonic inflammation, gastrointestinal hormones, interstitial cells of Cajal, irritable bowel syndrome, rikkunshito, TRP channels

## Abstract

**Purpose:**

Rikkunshito, a traditional Japanese herbal medicine, is used to improve gastrointestinal (GI) function. However, its mechanisms of action in irritable bowel syndrome (IBS) remain unclear. This study aimed to investigate the effects of Rikkunshito on colonic pacemaker activity, inflammation, GI hormones, pain-related behaviors, and transient receptor potential (TRP) channel activity in a zymosan-induced IBS mouse model.

**Methods:**

Rikkunshito composition was analyzed by UPLC. Pacemaker potentials were recorded in colonic interstitial cells of Cajal (ICCs) to examine ghrelin receptor–mediated Ca^2+^ signaling pathways. Zymosan-induced IBS was established in male C57BL/6 mice treated with Rikkunshito, sulfasalazine, or amitriptyline. Colonic pathology, inflammation, stool consistency, body weight, pain-related behaviors, gastrointestinal hormones, and epithelial barrier–related gene expression were evaluated. TRPV1, TRPV4, and TRPA1 channel activities were analyzed using whole-cell patch-clamp recordings in TRP-overexpressing HEK293T cells.

**Results:**

Rikkunshito depolarized colonic ICCs membranes and markedly suppressed pacemaker potential amplitude. These effects were mediated through ghrelin receptor–coupled G protein signaling and required extracellular Ca^2+^ influx and intracellular Ca^2+^ release via the PLC–IP_3_ pathway. In addition, MAPK signaling was involved, whereas PKC and PKA signaling were not. *In vivo*, Rikkunshito ameliorated colonic shortening, inflammation, diarrhea, and pain-related behaviors in zymosan-induced IBS mice. TNF-α expression and VIP levels were significantly reduced, and NPY levels were restored, while 5-HT showed a downward trend. Furthermore, Rikkunshito reinstated the expression of aquaporins and tight junction–related genes, indicating reinforcement of mucosal barrier integrity. In HEK293T cells, Rikkunshito inhibited TRPV1, slightly enhanced TRPV4, and robustly activated TRPA1 currents.

**Conclusion:**

Rikkunshito exerts protective effects in IBS by coordinately regulating colonic pacemaker activity via ghrelin receptor–dependent Ca^2+^ signaling, modulating neuroendocrine balance, improving epithelial barrier function, and differentially regulating TRP channel activity. These findings support the potential of Rikkunshito as a complementary therapeutic agent for IBS through integrated modulation of gut motility, inflammation, and visceral sensory pathways.

## 1 Introduction

Irritable bowel syndrome (IBS) is a disorder of gut–brain interaction characterized by recurrent abdominal pain accompanied by alterations in bowel habits and enhanced visceral sensitivity (Joshi et al., 2025; Mou et al., 2025). Its pathophysiology involves low-grade inflammation, dysregulated gastrointestinal (GI) motility, and imbalance of neuroendocrine factors, such as 5-HT (serotonin), vasoactive intestinal peptide (VIP), and neuropeptide Y (NPY), leading to impaired regulation of GI motility and sensory signaling (Aggeletopoulou et al., 2025a; Kovacs et al., 2025).

Interstitial cells of Cajal (ICCs) function as electrical pacemakers within the GI tract, producing rhythmic slow-wave activity that orchestrates smooth muscle contractility and intestinal motility (Choi et al., 2023; Lee et al., 2024). Impaired ICCs function has been implicated in IBS, leading to dysregulated intestinal peristalsis and visceral hypersensitivity (Chen et al., 2017). Emerging evidence suggests that abnormal ICCs pacemaker activity may disrupt coordinated motility patterns and contribute to diarrhea, constipation*,* and abdominal pain in IBS (Sanders et al., 2002; Chen et al., 2017; Choi et al., 2023; Lee et al., 2024). Modulation of ICCs activity therefore represents a promising therapeutic strategy to restore normal GI motility and sensory homeostasis (Sanders et al., 2002). However, the upstream signaling pathways regulating colonic ICCs pacemaker activity in relation to IBS remain incompletely understood.

Transient receptor potential (TRP) channels are broadly distributed in sensory neurons and GI tissues, where they function as non-selective cation channels (Alaimo and Rubert, 2019). These channels mediate nociception, mechanosensation, and chemosensation, playing a pivotal role in visceral pain and hypersensitivity, which are hallmarks of IBS (Beckers et al., 2017). Altered TRP channel activity has been linked to increased visceral sensitivity and abdominal pain in IBS patients, making them potential targets for pharmacological modulation.

Rikkunshito, a traditional Japanese Kampo medicine composed of eight herbal ingredients, has been used clinically to treat functional GI disorders, including dyspepsia (Masuy et al., 2020; Inokuchi et al., 2021). Previous studies suggest that Rikkunshito improves GI motility, modulates hormone secretion, and alleviates inflammation (Azushima et al., 2019; Kim et al., 2020). However, the precise mechanisms through which it exerts these effects in IBS—particularly with respect to colonic ICCs pacemaker regulation and sensory signaling pathways—remain unclear.

Zymosan-induced colitis in mice recapitulates several hallmarks of IBS, such as disrupted GI motility, low-grade inflammation, and enhanced visceral sensitivity, and is therefore widely used for therapeutic assessment (Choi et al., 2025a; Siting et al., 2025). In this study, we examined the impact of Rikkunshito on colonic morphology, inflammation, ICCs pacemaker activity, pain-related behaviors, GI hormone levels, and the expression of aquaporins and tight junction–related genes in zymosan-induced IBS mice. In addition, we examined the cellular mechanisms underlying colonic ICCs modulation, focusing on ghrelin receptor–dependent intracellular signaling pathways. Furthermore, we assessed its modulatory effects on TRPV1, TRPV4, and TRPA1 channel activity to explore potential mechanisms linking pacemaker regulation, epithelial barrier function, and visceral sensory modulation.

## 2 Methods

### 2.1 Preparation of Rikkunshito and reference standards

Rikkunshito (Tsumura & Co., Tokyo, Japan; Batch No. Y53981) consisted of eight constituent botanical drugs: *Atractylodes lancea* (Thunb.) DC. (2 g), *Panax ginseng* C.A.Mey. (2 g), *Pinellia ternata* (Thunb.) Makino (2 g), *Poria cocos* (Schw.) Wolf (2 g), *Ziziphus jujuba* Mill. (1 g), *Citrus unshiu* Marcov. (1 g), *Glycyrrhiza uralensis* Fisch. ex DC. (0.5 g), and *Zingiber officinale* Roscoe (0.25 g). The preparation is approved as a prescription Kampo medicine under the Japanese regulatory system. For quantitative analysis, reference plant metabolites were obtained from the following suppliers: 5-Hydroxymethyl-2-furfural, α-pinene, zingerone, and 6‴-feruloylspinosin from ChemFaces (Wuhan, China); glycyrrhizic acid, isoliquiritin, and liquiritin from Wako (Osaka, Japan); and L-tryptophan, naringin, naringenin, 6-gingerol, ginsenoside Rg1, and ginsenoside Rb1 from Sigma-Aldrich (St. Louis, MO, United States). Each metabolite was accurately weighed and dissolved in methanol to prepare a 1 μg/mL stock solution. Serial dilutions were then prepared in methanol to yield working standard solutions at 12.5, 25, 50, and 100 μg/mL. Calibration curves generated from these standard solutions showed excellent linearity, with all correlation coefficients (R^2^) exceeding 0.999. Botanical authentication was not performed in this study, as a standardized Kampo extract manufactured under pharmacopoeial quality control was used.

### 2.2 Ultra-performance liquid chromatography (UPLC) analytical conditions

The target plant metabolites were monitored at their respective optimal detection wavelengths as follows: 5-hydroxymethyl-2-furfural and α-pinene at 280 nm; L-tryptophan at 230 nm; glycyrrhizic acid and isoliquiritin at 254 nm; liquiritin at 276 nm; naringin, naringenin, 6-gingerol, zingerone, and 6‴-feruloylspinosin at 280 nm; and ginsenoside Rg1 and ginsenoside Rb1 at 203 nm. The mobile phase consisted of acetonitrile and water containing 0.1% formic acid. Samples were injected at a volume of 2 μL with a flow rate of 0.4 mL/min. Detailed analytical parameters for each plant metabolite are summarized in Table 1 (5-hydroxymethyl-2-furfural, α-pinene, L-tryptophan, glycyrrhizic acid, isoliquiritin, liquiritin, naringin, naringenin, 6-gingerol, zingerone, and 6‴-feruloylspinosin) and Table 2 (ginsenoside Rg1 and ginsenoside Rb1). All analytical procedures were performed according to previously validated methods reported in our previous study (Kim et al., 2020).

**TABLE 1:** Analytic conditions for detection of 5-Hydroxymethyl-2-furfural, α-Pinene, L-Tryptophan, Glycyrrhizic acid, Isoliquiritin, Liquiritin, Naringin, Naringenin, 6-Gingerol, Zingerone, and 6’’’-Feruloylspinosin in Rikkunshito.

Time (minute)	0.1% FA / water (%)	0.1% FA / acetonitrile (%)	Flow rate (ml/minute)
0	98	2	0.40
2.0	98	2	0.40
4.0	90	10	0.40
5.0	70	30	0.40
8.0	70	30	0.40
10.0	60	40	0.40
11.0	40	60	0.40
13.0	2	98	0.40
14.0	98	2	0.40
16.0	98	2	0.40

**TABLE 2:** Analytic conditions for detection of Ginsenoside Rg1 and Ginsenoside Rb1 in Rikkunshito.

Time (minutes)	Water (%)	Acetonitrile (%)	Flow rate (ml/ minutes)
0	85	15	0.40
1.0	85	15	0.40
14.0	70	30	0.40
15.0	68	32	0.40
16.0	60	40	0.40
17.0	45	55	0.40
19.0	45	55	0.40
21.0	10	90	0.40
22.0	10	90	0.40
23.0	85	15	0.40

### 2.3 Isolation and primary culture of ICCs

Colon were dissected from a total of fifty-eight ICR mice aged 3–7 days and the mucosal layer was carefully removed. The remaining smooth muscle tissues were incubated in Ca^2+^-free Hank’s solution and then enzymatically dissociated using a mixture of collagenase (Worthington Biochemical, Lakewood, NJ, United States), bovine serum albumin (Sigma-Aldrich, St. Louis, MO, United States), and trypsin inhibitor (Sigma-Aldrich, St. Louis, MO, United States). Isolated cells were maintained at 37 °C in a humidified 95% O_2_/5% CO_2_ incubator using smooth muscle growth medium (SMGM; Clonetics, San Diego, CA, United States) supplemented with 2% antibiotics/antimycotics (Gibco, Grand Island, NY, United States) and murine stem cell factor (5 ng/mL; Sigma-Aldrich, St. Louis, MO, United States). ICC clusters were used for electrophysiological experiments after 12 h of culture. All remaining procedures followed standard protocols widely applied in previous studies (Kim et al., 2020).

### 2.4 Animal model and experimental grouping

A total of thirty-five male C57BL/6 mice (21–26 g; Samtako Bio, Osan, Republic of Korea) were used for all experiments. Before initiating the study, the animals were given a 1-week adaptation period under standard housing conditions. Experimental colitis was induced by intrarectal administration of 0.1 mL zymosan solution (30 mg/mL; Sigma-Aldrich, St. Louis, MO, United States) using a flexible Zonde needle once daily for three consecutive days. The animals were then allocated into seven groups: a PBS-treated naïve group, a zymosan-exposed control group, three Rikkunshito-treated groups (100, 250, and 500 mg/kg), a sulfasalazine (SSZ, 30 mg/kg; Sigma-Aldrich) group, and an amitriptyline (AMT, 30 mg/kg; Sigma-Aldrich) group. AMT (Morgan et al., 2005) and SSZ (Pruzanski et al., 1997), both agents with established efficacy in clinical management of IBS, were used as positive treatments.

### 2.5 Assessment of fecal water content, colon morphology, stool consistency, and body weight

To evaluate zymosan-induced alterations in the colon, fecal water content was first measured to assess the diarrheal phenotype. Fresh fecal pellets were collected individually from mice at the end of the experimental period and immediately weighed to obtain the wet weight. The samples were then dried in an oven at 60 °C for 24 h to determine the dry weight. Fecal water content (%) was calculated as the difference between wet and dry weights divided by the wet weight, multiplied by 100. Colon length and weight were subsequently measured, with length determined as the distance from the cecum to the anus. Stool characteristics were assessed in a blinded manner by three independent researchers. Stool consistency was scored using a four-point system adapted from the Bristol Stool Scale: 0 (normal), 1 (moist), 2 (sticky), and 3 (diarrhea). Body weight was recorded on days 1, 4, 9, and 12 to monitor changes over time. In parallel, cumulative food intake was measured throughout the experimental period to evaluate feeding behavior.

### 2.6 Histopathological examination of the colon

For histological evaluation, colonic tissues were collected from each experimental group, fixed, paraffin-embedded, and sectioned. Sections were stained with hematoxylin and eosin (H&E) to examine overall tissue architecture. Histological images were acquired using a light microscope (Nikon, Japan) at 50× magnification.

### 2.7 *Quantification of* tumor necrosis factor *(TNF)-α expression in colonic tissue*

To quantify colonic TNF-α mRNA levels associated with colitis, total RNA was extracted from colon tissues using TRIzol reagent (Invitrogen, Waltham, MA, United States). The isolated RNA was reverse-transcribed into complementary DNA (cDNA) using an M-MLV reverse transcription kit (Promega, Madison, WI, United States). Quantitative real-time PCR was carried out with iTaq Universal SYBR Green Supermix (Bio-Rad, Hercules, CA, United States) and gene-specific primers on a StepOnePlus Real-Time PCR system (Applied Biosystems, Foster City, CA, United States).

### 2.8 Assessment of pain-related behaviors

The methods used to assess pain-related behaviors were conducted in accordance with previous studies (Laird et al., 2001). Observed behaviors included abdominal licking, full-body stretching, pressing the abdomen against the floor, and abdominal arching. To enhance the reliability of the data, two independent researchers recorded the frequency of these pain-related behaviors over a 10-min observation period.

### 2.9 Measurement of plasma GI hormones

Plasma GI hormone levels were assessed in three groups of animals: the naïve group, the zymosan-induced IBS group, and the group treated with 500 mg/kg Rikkunshito. Blood samples were collected from each mouse, and the concentrations of individual GI hormones were quantified using commercially available ELISA kits according to the manufacturers’ instructions. The ELISA kit for 5-HT was purchased from MyBioSource.com (San Diego, CA, United States). Kits for VIP and somatostatin were obtained from Abbkine Scientific Co., Ltd. (Atlanta, GA, United States). Ghrelin, GLP-1, and NPY ELISA kits were purchased from Antibodies.com (Cambridge, United Kingdom).

### 2.10 Quantitative real-time PCR analysis

Total RNA was isolated from colonic tissue using TRIzol reagent, following the protocol routinely applied in our laboratory (Choi et al., 2026). RNA integrity and concentration were assessed with a NanoDrop spectrophotometer. One microgram of purified RNA was converted to cDNA with a commercially available reverse transcription kit. Quantitative PCR was carried out on a StepOnePlus platform using SYBR Green chemistry and standard amplification parameters. Gene expression related to antioxidant activity was normalized to GAPDH, and relative transcriptional changes were determined by the 2^−ΔΔCt^ method. Primer information is listed in Supplementary Material 1. All reactions were performed in technical triplicates and included a minimum of three independent biological samples.

### 2.11 Electrophysiological recordings in ICCs and TRP-Transfected HEK293T Cells

Pacemaker potentials in ICCs were recorded using the whole-cell patch-clamp configuration at a temperature maintained between 30 °C and 33 °C. The compositions of both the pipette and bath solutions followed those described in our previous studies (Kim et al., 2020). To examine the effects of Rikkunshito on TRP channel activity, human TRPV1, TRPV4, and TRPA1 plasmids were transiently transfected into HEK293T cells. For visual verification of transfection efficiency, cells were co-transfected with 0.1 μg/mL pEGFP-N1. Patch-clamp recordings were performed 24 h after transfection. For TRP channel recordings, the same pipette and bath solution compositions used in earlier publications were employed (Choi et al., 2024). Electrophysiological signals were acquired using an Axopatch 200B amplifier (Axon Instruments, Foster City, CA, United States). Pipette resistance was adjusted to 3–5 MΩ, and recordings were accepted only when gigaseals greater than 1 GΩ were obtained. Data acquisition and analysis were conducted using pClamp software (Molecular Devices, San Jose, CA, United States) and Origin software version 2018 (OriginLab, Northampton, MA, United States).

### 2.12 Statistical analysis

Data are presented as the mean ± standard error (SE). Statistical differences among groups were evaluated using one-way analysis of variance (ANOVA) followed by Dunnett’s multiple comparison test. All analyses were performed using GraphPad Prism version 8 (GraphPad Software, San Diego, CA, United States), and a p-value less than 0.05 was considered statistically significant.

## 3 Results

### 3.1 Quantitative analysis of rikkunshito

**FIGURE 1:**
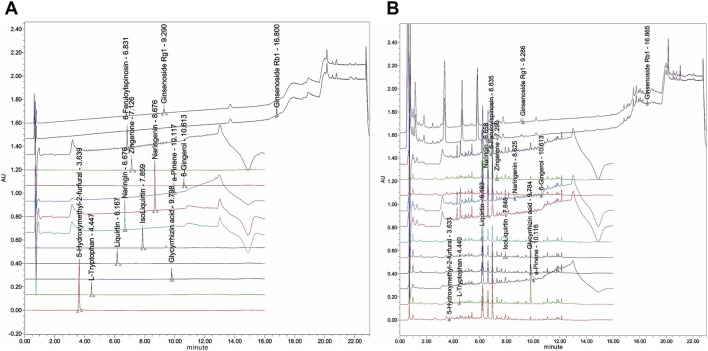
UPLC analysis of major constituents in Rikkunshito. **(A)** Chromatograms of the 13 reference standards used for quantification. **(B)** Representative UPLC chromatogram of Rikkunshito showing peaks corresponding to all analyzed constituents.

The levels of 13 major plant metabolites in Rikkunshito—including 5-hydroxymethyl-2-furfural, α-pinene, L-tryptophan, glycyrrhizic acid, isoliquiritin, liquiritin, naringin, naringenin, 6-gingerol, zingerone, 6‴-feruloylspinosin, ginsenoside Rg1, and ginsenoside Rb1—were quantified using UPLC. Their concentrations were determined based on calibration curves generated from the corresponding reference standards (Table 3; Figure 1). Method validation demonstrated acceptable linearity, precision, and reproducibility of the analytical method.

**TABLE 3:** Concentrations of 5-Hydroxymethyl-2-furfural, α-Pinene, L-Tryptophan, Glycyrrhizic acid, Isoliquiritin, Liquiritin, Naringin, Naringenin, 6-Gingerol, Zingerone, 6’’’-Feruloylspinosin, Ginsenoside Rg1 and Ginsenoside Rb1 in the Rikkunshito sample used for experiments as measured by UPLC.

Herbal components	Major constituents	Concentrations (mg/kg)
Atractylodes Rhizome White	5-Hydroxymethyl-2-furfural	0.142 ± 0.075
	L-Tryptophan,	7.386 ± 0.806
Pinelliae Tuber	α-Pinene	7.667 ± 0.958
Ginseng Radix	Ginsenoside Rg1,	13.571 ± 0.135
	Ginsenoside Rb1,	16.509 ± 0.287
Glycyrrhizae Radix et Rhizoma	Glycyrrhizic acid	132.111 ± 5.364
	Liquirtin	23.690 ± 0.421
	Isoliquirtin	8.532 ± 0.359
Citri Unshius Pericarpium	Naringin,	80.731 ± 7.652
	Naringenin	1.580 ± 0.185
Zingiber officinale Rosc	6-Gingerol,	3.659 ± 0.124
	Zingerone	15.278 ± 0.945
Zizyphi Fructus	6’’’-Feruloylspinosin	2.472 ± 0.712

### 3.2 Effects of Rikkunshito on pacemaker potentials in colonic ICCs via ghrelin receptor–G protein signaling

**FIGURE 2:**
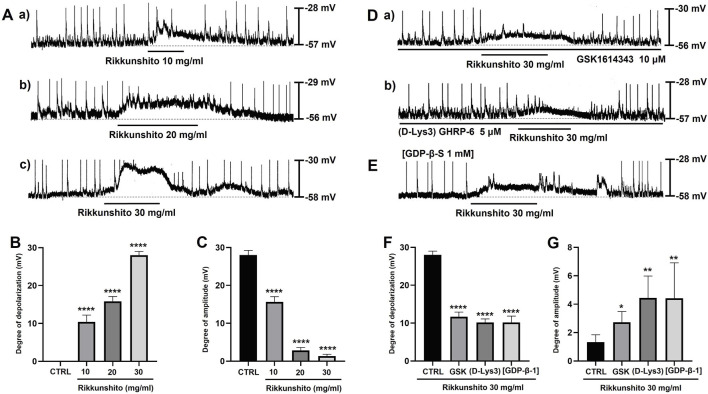
Modulation of pacemaker potentials in colonic ICCs by Rikkunshito and involvement of ghrelin receptor–G protein signaling. **(A**a–c**)** Representative pacemaker potential traces recorded after bath application of Rikkunshito (10–30 mg/mL). **(B)** Depolarization of resting membrane potential in a concentration-dependent manner. **(C)** Suppression of pacemaker potential amplitude following Rikkunshito treatment. **(D**a–b**)** Effects of ghrelin receptor antagonists on Rikkunshito-induced depolarization: **(D**a**)** GSK1614343 and **(D**b**)** (D-Lys3)-GHRP-6 both attenuated the depolarization of pacemaker potentials. **(E)** Representative traces showing the influence of intracellular GDP-β-S on Rikkunshito-induced depolarization. **(F,G)** Summary of changes in depolarization and amplitude in the presence of ghrelin receptor antagonists or GDP-β-S. Data are presented as mean ± SE. *p < 0.05, **p < 0.01, ****p < 0.0001 vs control group. CTRL, control; GSK, GSK1614343; (D-Lys3), (D-Lys3)-GHRP-6; GDP-β-S, guanosine 5′-[β-thio]diphosphate.

ICCs displayed spontaneous rhythmic depolarizations consistent with intrinsic pacemaker activity under control conditions (Figure 2). Bath application of Rikkunshito (10–30 mg/mL) produced a concentration-dependent depolarization of the resting membrane potential, together with a marked reduction in pacemaker potential amplitude (Figures 2Aa–c). The mean depolarization induced by Rikkunshito was 10.4 ± 1.8 mV at 10 mg/mL (n = 14; p < 0.0001), increased to 15.8 ± 1.3 mV at 20 mg/mL (n = 14; p < 0.0001), and reached 28.1 ± 1.0 mV at 30 mg/mL (n = 14; p < 0.0001) (Figure 2B). In parallel, pacemaker potential amplitude was significantly suppressed, decreasing from 15.7 ± 1.4 mV at 10 mg/mL to 2.8 ± 0.8 mV at 20 mg/mL and 1.3 ± 0.5 mV at 30 mg/mL (n = 14 for all groups; p < 0.0001) (Figure 2C). To determine whether ghrelin receptors are involved in Rikkunshito-induced modulation of pacemaker activity, ICCs were pretreated with the ghrelin receptor antagonists GSK1614343 (10 μM) or (D-Lys3)-GHRP-6 (5 μM) for 5 min. Neither antagonist alone altered basal pacemaker potentials; however, both significantly attenuated Rikkunshito-induced membrane depolarization and amplitude suppression (Figures 2Da,b). In the presence of GSK1614343, Rikkunshito-induced depolarization was reduced to 11.7 ± 1.2 mV (n = 8; p < 0.0001), while pacemaker potential amplitude was maintained at 2.7 ± 0.8 mV (n = 8; p < 0.05). Similarly, pretreatment with (D-Lys3)-GHRP-6 resulted in a depolarization of 10.2 ± 1.0 mV (n = 8; p < 0.0001) and an amplitude of 4.4 ± 1.6 mV (n = 8; p < 0.01) (Figures 2F,G). Because the ghrelin receptor is coupled to G proteins, the involvement of G protein signaling was further examined using intracellular application of GDP-β-S (1 mmol/L). Under these conditions, Rikkunshito induced only a modest depolarization of 10.1 ± 1.7 mV (n = 8; p < 0.0001) and was associated with a partial recovery of pacemaker potential amplitude to 4.4 ± 2.5 mV (n = 8; p < 0.01) (Figures 2E–G). Taken together, these data demonstrate that Rikkunshito regulates colonic ICCs pacemaker potentials via activation of ghrelin receptors coupled to G protein signaling pathways.

### 3.3 Effects of Ca^2+^ mobilization, PLC–IP_3_ signaling, and PKC/MAPK inhibition on Rikkunshito-induced depolarization of pacemaker potentials in colonic ICCs

**FIGURE 3:**
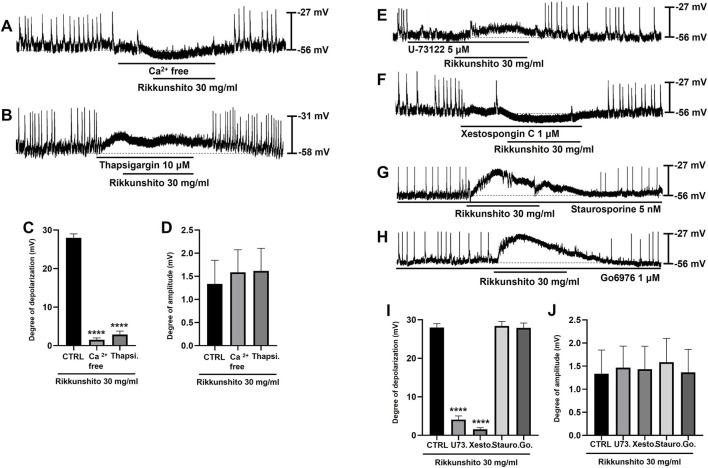
Roles of Ca^2+^ signaling, PLC–IP_3_ pathways, and PKC in Rikkunshito-induced modulation of colonic ICCs pacemaker potentials. **(A)** Removal of external Ca^2+^ abolished pacemaker potentials and prevented depolarization by Rikkunshito. **(B)** Thapsigargin treatment similarly blocked pacemaker potentials and inhibited Rikkunshito-induced depolarization. **(C,D)** Summary of Rikkunshito-induced changes in depolarization and amplitude under Ca^2+^-free or thapsigargin-treated conditions. **(E)** Pretreatment with the PLC inhibitor U-73122 suppressed pacemaker potentials and blocked Rikkunshito-mediated depolarization. **(F)** IP_3_ receptor blockade by xestospongin C also prevented depolarization induced by Rikkunshito. **(G,H)** Depolarization persisted in the presence of PKC inhibitors: staurosporine (broad-spectrum) and Go6976 (Ca^2+^-dependent PKC α/β), indicating a PKC-independent mechanism. **(I,J)** Summary of depolarization and amplitude in the presence of PLC/IP_3_ receptor inhibitors or PKC blockade. Data are expressed as mean ± SE. ****p < 0.0001 vs control group. CTRL, control; Thapsi., thapsigargin; U73., U-73122; Xesto., Xestospongin C; Stauro., staurosporine; Go., Go6976.

**FIGURE 4:**
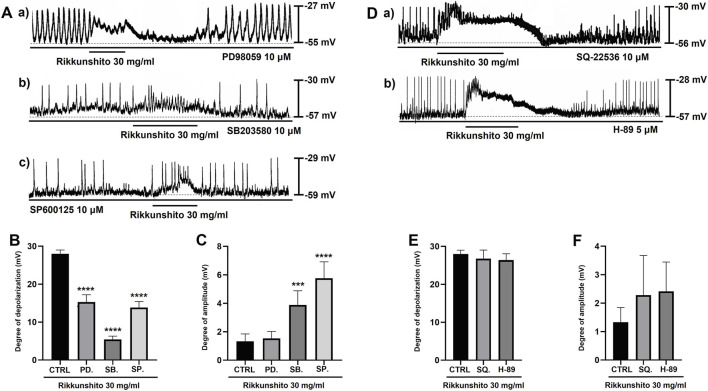
Effects of MAPK and PKA inhibitors in Rikkunshito-induced modulation of colonic ICCs pacemaker potentials. **(A)** Representative traces showing the effects of MAPK inhibitors, including **(A)** PD98059, **(B)** SB203580, and **(C)** SP600125 on Rikkunshito-induced depolarization. **(B,C)** Summary of depolarization and amplitude in the presence of MAPK inhibitors. **(D)** Representative traces showing the effects of PKA inhibitors, **(A)** SQ-22536 and **(B)** H-89. **(E,F)** Summary of depolarization and amplitude in the presence of PKA inhibitors. Data are expressed as mean ± SE. ***p < 0.001 and ****p < 0.0001 vs control group. CTRL, control; PD., PD98059; SB., SB203580; SP., SP600125; SQ., SQ-22536.

Because coordinated intracellular Ca^2+^ dynamics are essential for the generation of GI pacemaker activity, the contribution of both extracellular Ca^2+^ influx and intracellular Ca^2+^ release to Rikkunshito-induced depolarization was examined in colonic ICCs. When pacemaker potentials were recorded under external Ca^2+^-free conditions, Rikkunshito failed to induce membrane depolarization (Figure 3A). Similarly, inhibition of endoplasmic reticulum Ca^2+^-ATPase with thapsigargin completely abolished the depolarizing effect of Rikkunshito (Figure 3B). Under Ca^2+^-free conditions or in the presence of thapsigargin, the mean depolarization was limited to 1.5 ± 0.5 mV and 2.9 ± 0.9 mV, respectively (n = 8; both p < 0.0001; Figure 3C), and pacemaker potential amplitude was markedly reduced to 1.5 ± 0.5 mV and 1.6 ± 0.4 mV, respectively (n = 8; Figure 3D). Given that G protein signaling commonly regulates phospholipid-dependent pathways, the involvement of phospholipase C (PLC) in Rikkunshito-induced depolarization was next evaluated. Pretreatment of ICCs with the PLC inhibitor U-73122 (5 μM) strongly suppressed basal pacemaker activity and prevented Rikkunshito-induced depolarization (Figure 3E). Consistent with this finding, blockade of inositol 1,4,5-triphosphate (IP_3_) receptors with xestospongin C (1 μM) also abolished the depolarizing response to Rikkunshito (Figure 3F). In the presence of U-73122 or xestospongin C, the mean depolarization was reduced to 4.1 ± 0.9 mV and 1.6 ± 0.4 mV, respectively (Figure 3I), while pacemaker potential amplitude remained low at 1.5 ± 0.5 mV and 1.4 ± 0.5 mV, respectively (Figure 3J). In contrast, inhibition of protein kinase C (PKC) signaling did not attenuate the effects of Rikkunshito on pacemaker potentials. In the presence of the broad-spectrum PKC inhibitor staurosporine (5 nM) or the Ca^2+^-dependent PKCα/β inhibitor Go6976 (1 μM), Rikkunshito continued to induce robust membrane depolarization (Figures 3G,H). The mean depolarization under these conditions was 28.4 ± 1.2 mV with staurosporine and 27.9 ± 1.3 mV with Go6976 (Figure 3I), accompanied by marked suppression of pacemaker potential amplitude to 1.6 ± 0.5 mV and 1.4 ± 0.5 mV, respectively (Figure 3J). To further investigate the involvement of additional signaling pathways, MAPK and PKA inhibitors were applied prior to Rikkunshito treatment. Pretreatment with MAPK inhibitors, including PD98059 (ERK pathway inhibitor), SB203580 (p38 MAPK inhibitor), and SP600125 (JNK inhibitor), significantly attenuated Rikkunshito-induced depolarization of ICCs pacemaker potentials (Figures 4A–C). In contrast, pretreatment with PKA inhibitors, SQ-22536, an adenylyl cyclase inhibitor that reduces cAMP production, and H-89, a selective protein kinase A (PKA) inhibitor, did not significantly affect the Rikkunshito-induced responses (Figures 4D–F). Taken together, these findings indicate that Rikkunshito-induced depolarization of pacemaker potentials in colonic ICCs requires both extracellular and intracellular Ca^2+^ signaling and depends on the PLC–IP_3_ pathway. MAPK signaling is also involved, whereas PKC and PKA signaling are not.

### 3.4 Rikkunshito ameliorates fecal water content changes, colonic dysfunction, and weight loss in a zymosan-induced IBS mice

**FIGURE 5:**
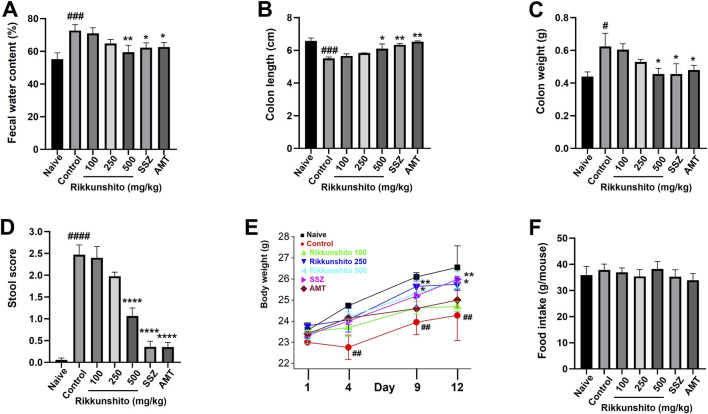
Effects of Rikkunshito on fecal water content, colon morphology, stool characteristics, and body weight in zymosan-induced IBS mice. **(A)** Fecal water content. **(B)** Colon length. **(C)** Colon weight. **(D)** Stool consistency scores. **(E)** Body weight changes. **(F)** Cumulative food intake. Rikkunshito restored fecal water content, colon parameters, improved stool scores, and prevented weight loss. Data are shown as mean ± SE. #p < 0.05, ###p < 0.001 and ####p < 0.0001 vs naive group. *p < 0.05, **p < 0.01, and ****p < 0.0001 vs control group.

We evaluated fecal water content, colonic morphology, stool characteristics, and body weight changes in zymosan-treated mice following Rikkunshito administration. Fecal water content was significantly increased in zymosan-induced IBS mice compared with naïve mice (55.2% ± 3.8% vs. 72.7% ± 3.7%, *p* < 0.001), indicating a diarrheal phenotype. Rikkunshito treatment significantly reduced fecal water content compared with the zymosan control group (Figure 5A). Zymosan markedly shortened colon length and increased colon weight compared with naïve mice, confirming successful induction of colitis. Rikkunshito treatment restored colon length toward normal values and reduced the zymosan-induced increase in colon weight in a dose-dependent manner (Figures 5B,C). Stool scores were also significantly elevated in the zymosan control group, indicating severe diarrhea-like symptoms; however, Rikkunshito markedly lowered stool scores, with the greatest improvement observed at 500 mg/kg (Figure 5D). In addition to GI abnormalities, zymosan administration led to progressive body weight loss on days 4, 9, and 12. Rikkunshito effectively suppressed this loss, particularly at 500 mg/kg, where body weight was maintained at levels close to those of naïve mice (Figure 5E). Notably, food intake was comparable among all groups, indicating that the protective effects of Rikkunshito were not associated with changes in feeding behavior (Figure 5F). Taken together, these results demonstrate that Rikkunshito ameliorates zymosan-induced colonic alterations, reduces diarrhea symptoms, and prevents weight loss without affecting food consumption.

### 3.5 Rikkunshito attenuates colonic inflammation and pain-related behaviors in zymosan-induced IBS mice

**FIGURE 6:**
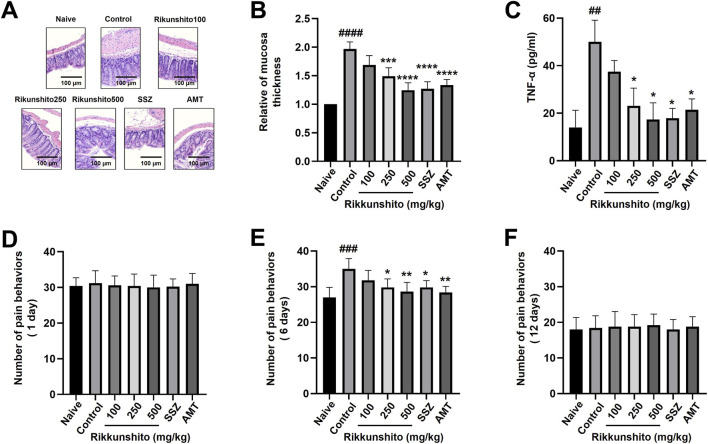
Rikkunshito attenuates colonic inflammation and pain-related behaviors in zymosan-induced IBS mice. **(A)** Representative H&E-stained colon sections. **(B)** Quantification of colon thickness. **(C)** TNF-α mRNA expression in colon tissue. **(D–F)** Pain-related behaviors on days 1, 6, and 12. Rikkunshito reduced inflammatory changes and nociceptive responses. Data are shown as mean ± SE. ##p < 0.01, and ###p < 0.001 vs naive group. *p < 0.05, and **p < 0.01 vs control group.

Inflammatory alterations were evident in the colons of zymosan-treated mice. H&E staining revealed substantial histopathological disruption in the zymosan control group, including pronounced thickening of the colonic wall compared with naïve mice. Rikkunshito administration attenuated these structural abnormalities in a dose-dependent manner, with tissue thickness approaching that of naïve animals (Figures 6A,B). Colon tissues collected on day 12 were further analyzed for TNF-α expression, an established molecular marker of inflammation. Zymosan markedly elevated TNF-α levels relative to naïve mice, whereas 250 and 500 mg/kg Rikkunshito significantly reduced this increase (Figure 6C), confirming its anti-inflammatory effects. We additionally evaluated pain-related behaviors associated with zymosan-induced IBS. No pain-related behaviors were observed on day 1 (Figure 6D). However, by day 6, zymosan-treated mice displayed characteristic nociceptive responses, including abdominal licking, abdominal pressing, whole-body stretching, and abdominal grabbing. At this time point, pairwise statistical analysis revealed that pain-related behaviors were significantly increased in the zymosan control group compared with naïve mice (naïve: 27.1 ± 2.8; control: 35.0 ± 2.9, *p* < 0.001). Rikkunshito at 100 mg/kg showed no significant difference compared with the control group, whereas 250 mg/kg and 500 mg/kg significantly reduced pain-related behaviors (*p* < 0.05 and *p* < 0.01, respectively). Both positive controls, SSZ and AMT, also significantly reduced pain-related behaviors compared with the control group (*p* < 0.05 and *p* < 0.01, respectively). Importantly, no significant differences were observed between Rikkunshito-treated groups and either SSZ or AMT, and no difference was detected between SSZ and AMT (Figure 6E). These nociceptive behaviors were no longer evident by day 12 (Figure 6F), indicating resolution of the zymosan-induced pain phenotype over time. Taken together, these findings demonstrate that Rikkunshito not only mitigates colonic inflammation but also alleviates pain-related responses in zymosan-induced IBS mice.

### 3.6 Effects of Rikkunshito on GI hormone profiles and epithelial barrier–related gene expression in zymosan-induced IBS mice

**FIGURE 7:**
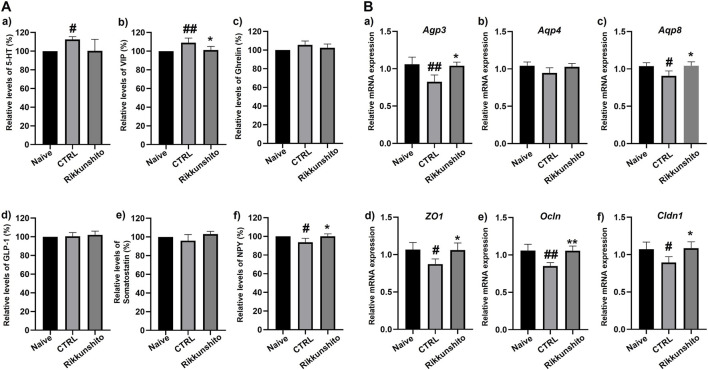
Effects of Rikkunshito on gastrointestinal hormone levels and epithelial barrier–related gene expression in zymosan-induced IBS mice. **(A)** Plasma concentrations of gastrointestinal hormones in zymosan-treated mice: (a) 5-HT, (b) VIP, (c) Ghrelin, (d) GLP-1, (e) Somatostatin, and (f) NPY. Rikkunshito significantly reduced VIP elevation and restored NPY levels. **(B)** Gene expression analysis in colonic tissues: (a–c) Aquaporins (*Aqp3*, *Aqp4*, *Aqp8*) were downregulated by zymosan and restored toward normal levels by Rikkunshito treatment. (d–f) Tight junction–related genes (*ZO1*, *Occludin*, *Claudin1*) were suppressed in the zymosan control group but significantly upregulated following Rikkunshito administration, indicating improved epithelial barrier function. Data are expressed as mean ± SE. #p < 0.05, ##p < 0.01 vs naïve group; *p < 0.05, **p < 0.01 vs control group. CTRL, control.

Administration of zymosan induced marked alterations in GI hormone profiles in the IBS mouse model (Figure 7A). Serotonin (5-HT) levels were elevated following zymosan treatment; although Rikkunshito administration did not result in a statistically significant reduction, a decreasing trend was observed compared with the untreated IBS group (Figure 7Aa). Vasoactive intestinal peptide (VIP) levels were significantly increased in zymosan-treated mice, whereas Rikkunshito treatment significantly reduced VIP expression (p < 0.05; Figure 7Ab). In contrast, the levels of ghrelin, glucagon-like peptide-1 (GLP-1), and somatostatin were not significantly affected by either zymosan exposure or Rikkunshito treatment, remaining comparable across experimental groups (Figures 7Ac–e). Neuropeptide Y (NPY) expression was markedly reduced in the zymosan-induced IBS group; however, Rikkunshito administration significantly restored NPY levels compared with the untreated IBS group (p < 0.05; Figure 7Af). In parallel with hormonal alterations, zymosan challenge significantly disrupted the expression of genes involved in water transport and epithelial barrier function in colonic tissues (Figure 7B). The mRNA expression levels of aquaporins *Aqp3*, *Aqp4*, and *Aqp8* were substantially downregulated in zymosan-treated mice, whereas Rikkunshito administration restored the expression of these water channel genes toward control levels (Figures 7Ba–c). Similarly, zymosan exposure resulted in decreased expression of key tight junction–associated genes, including *ZO1*, *Claudin1*, and *Occludin*. Treatment with Rikkunshito significantly upregulated the expression of these tight junction components compared with the IBS group, indicating recovery of epithelial barrier–related gene expression (Figures 7Bd–f). Taken together, these findings demonstrate that Rikkunshito selectively modulates zymosan-induced neuroendocrine disturbances—particularly VIP and NPY—and concurrently improves molecular markers associated with water transport and epithelial barrier integrity in IBS mice.

### 3.7 Rikkunshito selectively modulates TRP channel activity in HEK293T cell

**FIGURE 8:**
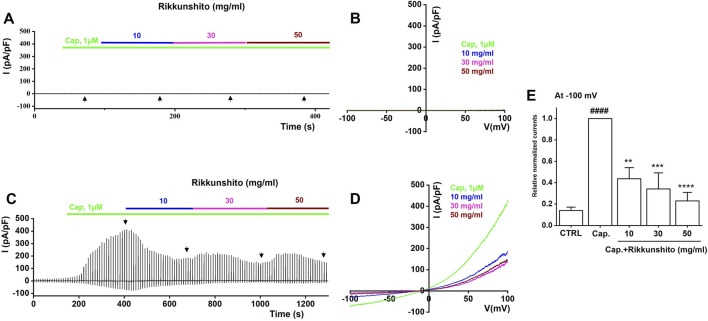
Modulatory effects of Rikkunshito on TRPV1 currents in TRPV1-expressing HEK293T cells. **(A,B)** No detectable inward or outward currents were observed in mock-transfected cells under capsaicin and Rikkunshito treatment. **(C)** Representative traces showing capsaicin-activated TRPV1 currents in TRPV1-transfected cells by Rikkunshito. **(D)** I–V relationships recorded in the presence and absence of Rikkunshito. **(E)** Summary of current amplitudes measured at −100 mV. Rikkunshito significantly reduced TRPV1-mediated currents in a concentration-dependent manner. ####p < 0.0001 vs control group. **p < 0.01, and ***p < 0.001 vs capsaicin group.

**FIGURE 9:**
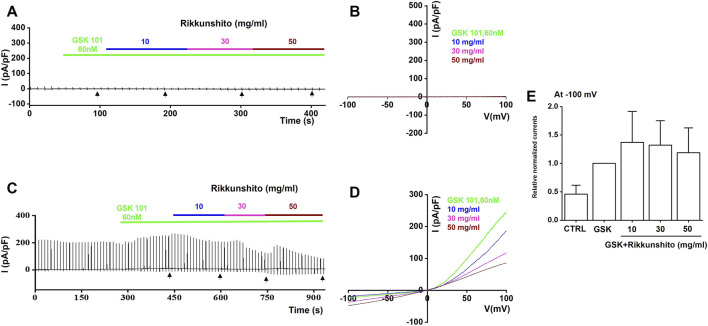
Effects of Rikkunshito on TRPV4 currents in TRPV4-expressing HEK293T cells. **(A,B)** No detectable inward or outward currents were observed in mock-transfected cells under GSK101 and Rikkunshito treatment. **(C)** Representative traces showing GSK101-activated TRPV4 currents in TRPV4-transfected cells by Rikkunshito. **(D)** I–V relationships recorded in the presence and absence of Rikkunshito. **(E)** Summary of current amplitudes measured at −100 mV. Rikkunshito slightly increased TRPV4 currents without statistical significance.

**FIGURE 10:**
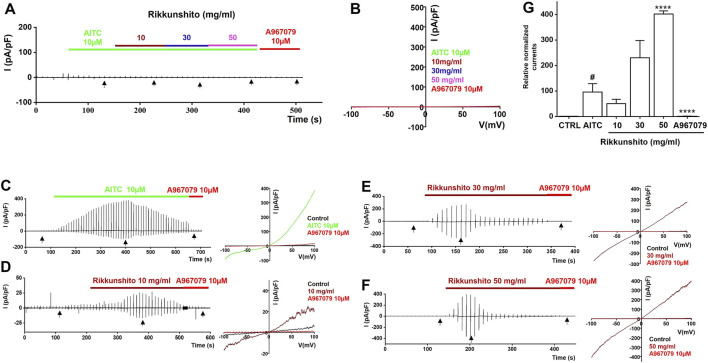
Rikkunshito activates TRPA1 currents in TRPA1-expressing HEK293T cells. **(A,B)** No detectable inward or outward currents were observed in mock-transfected cells under AITC and Rikkunshito treatment. **(C)** AITC-induced TRPA1 currents and their inhibition by A967079. **(D–F)** Dose-dependent enhancement of TRPA1 currents by Rikkunshito. **(G)** Potentiation fully blocked by the TRPA1 antagonist A967079, confirming TRPA1-mediated activation. #p < 0.05 vs control group. ****p < 0.0001 vs AITC group.

To investigate the modulatory effects of Rikkunshito on TRP channels, we performed whole-cell patch-clamp recordings in HEK293T cells overexpressing TRPV1, TRPV4, or TRPA1. Current–voltage (I–V) relationships were generated using a ramp pulse from −100 mV to 100 mV under control conditions and in the presence of 10, 30, or 50 mg/mL Rikkunshito. In mock-transfected cells, neither specific agonists (capsaicin for TRPV1, GSK1016790A for TRPV4, or AITC for TRPA1) nor Rikkunshito alone elicited measurable currents (Figures 8A,B, 9A,B, 10A,B), confirming that observed responses were specific to TRP channel overexpression. In TRPV1-overexpressing cells, capsaicin robustly activated TRPV1 currents, which were dose-dependently inhibited by Rikkunshito at −100 mV (Figures 8C–E). Using identical experimental conditions, GSK1016790A induced TRPV4 currents in TRPV4-overexpressing cells, and Rikkunshito treatment caused a tendency toward increased currents, although this effect was not statistically significant (Figures 9C–E). Finally, in TRPA1-overexpressing cells, AITC strongly activated TRPA1 currents, which were blocked by the TRPA1-specific inhibitor A967079. Remarkably, Rikkunshito at all tested concentrations (10, 30, and 50 mg/mL) enhanced TRPA1 currents, and this enhancement was fully suppressed by A967079, indicating that Rikkunshito acts as a TRPA1 activator (Figures 10C–G). Taken together, these findings demonstrate that Rikkunshito exerts selective modulatory effects on TRP channels: it inhibits TRPV1, slightly potentiates TRPV4, and functions as an activator of TRPA1 in overexpressing HEK293T cells. This coordinated regulation suggests that Rikkunshito may differentially modulate TRP-mediated signaling pathways relevant to IBS.

## 4 Discussion

Rikkunshito, a traditional Japanese herbal formulation, has been shown to exert diverse pharmacological effects, including the regulation of GI motility, anti-inflammatory effects, regulation of neuroendocrine pathways, and enhancement of visceral pain tolerance (Azushima et al., 2019; Kim et al., 2020; Masuy et al., 2020; Inokuchi et al., 2021). In this study, we demonstrated that Rikkunshito exerts multifaceted protective effects in a zymosan-induced mouse model of IBS, modulating colonic morphology, inflammation, pain-related behaviors, GI hormone levels, and ICCs pacemaker activity. Notably, our findings extend previous observations by providing mechanistic evidence that Rikkunshito directly regulates colonic ICCs pacemaker activity through defined intracellular signaling pathways. These results offer novel perspectives on how traditional herbal medicines modulate GI function and visceral sensitivity.

Zymosan-induced colitis in mice recapitulates several key features of IBS, including colonic wall thickening, disrupted stool consistency, weight loss, and heightened pain-related behaviors (Choi et al., 2025a; Siting et al., 2025). Our results indicate that Rikkunshito treatment restored fecal water content and colonic morphology (Figures 5A–C), reduced tissue thickening (Figures 6A,B), improved stool consistency (Figure 5D), and prevented weight loss in a dose-dependent manner (Figure 5E). These results are consistent with prior clinical and preclinical studies indicating that Rikkunshito contributes to GI homeostasis and helps maintain mucosal integrity. The observed normalization of TNF-α expression further highlights its anti-inflammatory potential (Figure 6C), consistent with earlier reports of Rikkunshito-mediated suppression of pro-inflammatory cytokines.

**FIGURE 11:**
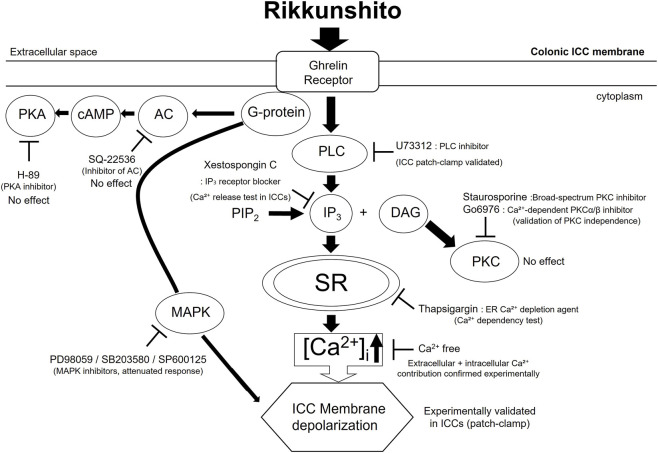
Schematic representation of the proposed mechanism of Rikkunshito action in colonic ICCs. Rikkunshito binds to ghrelin receptors on colonic ICCs, activating G protein–dependent signaling. This triggers PLC–IP_3_–mediated intracellular Ca^2+^ release and extracellular Ca^2+^ influx, leading to depolarization of pacemaker potentials and suppression of potential amplitude. PKC signaling is not involved, as indicated by the persistence of depolarization in the presence of PKC inhibitors (staurosporine or Go6976). In addition, MAPK signaling contributes to Rikkunshito-induced depolarization, as demonstrated by the attenuation of responses following treatment with specific MAPK inhibitors (PD98059, SB203580, and SP600125), whereas PKA signaling is not involved, as pretreatment with SQ-22536 and H-89 did not affect the response. All pharmacological interventions were validated in ICCs using patch-clamp recordings of pacemaker potentials. The diagram indicates experimentally confirmed signaling nodes and pharmacological inhibition sites. AC, Adenylate Cyclase; PKA, Protein Kinase A; MAPK, Mitogen-Activated Protein Kinase; PLC, Phospholipase C; PKC, Protein Kinase C; ICCs, Interstitial cells of Cajal.

ICCs function as GI pacemakers, producing rhythmic slow-wave electrical activity that synchronizes smooth muscle contractions (Sanders, 2019; Choi et al., 2023; Lee et al., 2024). Dysfunction of ICCs is implicated in abnormal motility and visceral hypersensitivity in IBS (Chen et al., 2017; Sanders et al., 2002). In this study, Rikkunshito induced depolarization of the resting membrane potential in ICCs while reducing the amplitude of pacemaker potentials in a concentration-dependent manner (Figures 2A–C), suggesting that it can modulate ICCs excitability and thereby regulate gut motility. Pharmacological analyses further revealed that this modulation depends on ghrelin receptor activation and downstream G protein–coupled signaling, requiring both extracellular Ca^2+^ influx and intracellular Ca^2+^ release via the PLC–IP_3_ pathway, while being independent of PKC signaling (Figures 2D–G; Figure 3). In addition, inhibition of MAPK signaling using PD98059, SB203580, and SP600125 significantly attenuated Rikkunshito-induced depolarization (Figures 4A–C), whereas PKA inhibition with SQ-22536 and H-89 had no significant effect (Figures 4D–F), indicating that MAPK, but not PKA, contributes to this response. Guided by the present findings and previous studies on ghrelin signaling and ICCs pacemaker activity (Cong et al., 2010; Kim et al., 2020; Inokuchi et al., 2021; Yakabi et al., 2024; Choi et al., 2025b), we propose a schematic model illustrating the mechanism by which Rikkunshito modulates pacemaker activity in colonic ICCs (Figure 11) - Rikkunshito activates ghrelin receptors, leading to depolarization of pacemaker potentials via G protein–coupled activation of the PLC–IP_3_–Ca^2+^ signaling pathway, with additional involvement of MAPK signaling in ICCs. These results indicate that Rikkunshito regulates colonic ICCs pacemaker activity through a specific Ca^2+^-dependent signaling cascade rather than nonspecific membrane effects. This modulation may contribute to the observed improvements in stool consistency and overall colonic function.

GI hormones are crucial mediators of gut motility and visceral sensation (Baynes et al., 2006; Vincent et al., 2008). Zymosan treatment elevated 5-HT and VIP levels while reducing NPY expression, reflecting a dysregulated neuroendocrine environment that contributes to motility disturbances and abdominal pain (Figure 7). Rikkunshito selectively normalized VIP and NPY levels, though its effect on 5-HT was not statistically significant. These data suggest that Rikkunshito exerts partial but targeted modulation of hormone signaling, which may underlie its beneficial effects on both motility and visceral sensitivity. Given the known interactions between ghrelin signaling, ICCs function, and enteric neuroendocrine regulation, coordinated modulation of these pathways may amplify the therapeutic effects of Rikkunshito on gut motility.

In addition, Rikkunshito treatment also restored the expression of aquaporins (*Aqp3*, *Aqp4*, and *Aqp8*) that were downregulated in zymosan-induced IBS-D mice, suggesting that its therapeutic effects may involve normalization of water transport and maintenance of mucosal hydration (Figures 7Ba–c). In parallel, Rikkunshito significantly upregulated the expression of tight junction–related genes, including *ZO-1*, *Claudin-1*, and *Occludin*, which were suppressed in the same zymosan-induced IBS-D model (Figures 7Bd–f). This coordinated regulation of water channels and epithelial barrier components likely contributes to the improvement of intestinal barrier integrity and alleviation of diarrhea symptoms. It is important to recognize that alterations in aquaporin and tight junction expression are highly model- and phenotype-dependent. For instance, in IBS-C models, AQP3 is often upregulated, whereas in IBS-D or other secretory/diarrheal models, *Aqp3*, *Aqp4*, and *Aqp8* are commonly downregulated (Hardin et al., 2004; Chao and Zhang, 2017; Zhu et al., 2023). Similarly, tight junction proteins are frequently diminished in IBS-D models due to increased epithelial permeability and inflammatory stress, whereas their regulation may differ in IBS-C or stress-induced models (Zhao et al., 2022; Guo et al., 2023). These differences likely reflect the underlying pathophysiology, including variations in intestinal motility, epithelial barrier disruption, inflammatory status, and osmotic water handling (Hanning et al., 2021). Moreover, discrepancies among studies may arise from differences in experimental design, such as the type of IBS model (chemical, stress, or infection), tissue sampling location, timing of analysis, and whether mRNA or protein levels were assessed (Aggeletopoulou et al., 2025b). Therefore, our findings indicate that Rikkunshito exerts protective effects in IBS-D by simultaneously restoring aquaporin expression and reinforcing tight junction integrity, thereby improving both water transport and epithelial barrier function in the colon.

Pain-related behaviors in IBS are mediated in part by TRP channels expressed in sensory neurons and GI tissues. TRPV1, TRPV4, and TRPA1 have been implicated in nociceptive signaling and visceral hypersensitivity. Our patch-clamp studies in HEK293T cells revealed that Rikkunshito selectively modulates TRP channel activity: it inhibited TRPV1 currents (Figure 8), slightly potentiated TRPV4 currents (Figure 9), and acted as an activator of TRPA1 (Figure 10). These differential effects suggest that Rikkunshito may fine-tune TRP-mediated sensory signaling, suppressing pro-nociceptive TRPV1 activity while modulating other channels involved in mechanosensation and chemosensation. Given that ICCs pacemaker activity influences smooth muscle tone and mechanical stimuli within the gut wall, coordinated regulation of ICCs excitability and TRP channel signaling may collectively reduce visceral hypersensitivity. Although these electrophysiological results were obtained *in vitro*, they provide a plausible mechanistic explanation for the reduction of pain-related behaviors observed *in vivo* (Figures 6D–F).

A key conceptual link between the ICC and TRP channel findings is the central role of Ca^2+^ signaling in GI excitability. In ICCs, Rikkunshito modulated pacemaker activity through a ghrelin receptor–dependent G protein–PLC–IP_3_ pathway, leading to intracellular Ca^2+^ mobilization. In parallel, TRP channels such as TRPV1, TRPV4, and TRPA1 are well-established Ca^2+^-permeable channels that regulate visceral sensory signaling and epithelial excitability. Accordingly, the TRP channel experiments were designed to determine whether Rikkunshito also influences Ca^2+^ entry pathways at the plasma membrane level, complementing its effects on intracellular Ca^2+^ release mechanisms observed in ICCs. The observed differential modulation of TRPV1 inhibition, TRPV4 activation, and TRPA1 activation suggests that Rikkunshito exerts a broad regulatory effect on Ca^2+^-dependent excitability systems. Collectively, these findings support a unified mechanism in which Rikkunshito regulates GI function through coordinated modulation of both GPCR-mediated intracellular Ca^2+^ signaling in pacemaker cells and TRP channel–mediated Ca^2+^ influx pathways involved in sensory and epithelial responses.

SSZ and AMT were included as two positive controls representing distinct therapeutic strategies for IBS. SSZ primarily reflects an anti-inflammatory approach targeting intestinal immune activation, whereas AMT is a neuromodulatory agent widely used for visceral pain modulation and gut–brain axis regulation. Although both agents demonstrated broadly comparable effects in the present model, their inclusion provides complementary reference points for inflammation- and pain-related aspects of IBS pathophysiology. In this context, Rikkunshito should be interpreted as a multi-target botanical drug rather than a single-mechanism therapeutic agent. Unlike SSZ and AMT, which primarily act on specific pathological domains, Rikkunshito simultaneously modulates gastrointestinal motility, inflammatory responses, epithelial barrier integrity, neuroendocrine signaling, and TRP channel activity. This integrated regulatory profile may provide a broader systems-level modulation of IBS pathophysiology, which is not fully captured by conventional single-target pharmacotherapies. Therefore, the inclusion of both SSZ and AMT serves not only as comparative pharmacological benchmarks but also as a framework to contextualize the multi-modal actions of Rikkunshito within established IBS treatment paradigms.

Taken together, our findings indicate that Rikkunshito acts on multiple levels of gut physiology. It mitigates inflammation, normalizes GI hormone profiles, modulates ICCs pacemaker activity, and regulates TRP channel function. Importantly, the ghrelin receptor–dependent Ca^2+^ signaling in colonic ICCs identified in this study provides a mechanistic link between traditional herbal medicine and enteric pacemaker regulation. This multimodal action likely contributes to the overall improvement of colonic function and visceral pain in IBS. The coordinated regulation of ICCs activity and TRP channel signaling further suggests a convergence of enteric motor and sensory mechanisms in the therapeutic effects of Rikkunshito.

Traditional herbal medicines have been valued for centuries for their ability to treat complex and multifactorial diseases (Shao et al., 2025; Xie et al., 2025). Unlike conventional pharmaceuticals that typically target a single molecular pathway, traditional formulations such as Rikkunshito can act on multiple physiological systems simultaneously (Gureje et al., 2015; Steel et al., 2020). In the context of GI disorders, this multimodal action offers significant advantages, as IBS and other functional gut diseases involve a combination of motility disturbances, neuroendocrine dysregulation, immune activation, and visceral hypersensitivity (Bahrami et al., 2016; Abalo et al., 2025). Our results provide empirical support for the efficacy of Rikkunshito in modulating these interconnected pathways, highlighting the unique strengths of traditional herbal therapies in managing multifactorial conditions. In recent years, there has been growing interest in traditional medicine worldwide, driven by increasing recognition of its therapeutic potential and safety profile (Leonti and Casu, 2013; van der Kooy and Sullivan, 2013). Scientific validation of traditional formulations through modern experimental techniques, including electrophysiology, hormone profiling, and molecular biology, is crucial for integrating these therapies into contemporary medical practice (Ijaz et al., 2024; Bashir, 2025). Our study contributes to this trend by combining *in vivo* IBS models with *in vitro* mechanistic analyses, elucidating how Rikkunshito modulates ICCs function and TRP channel activity to mediate GI benefits. Looking forward, the integration of traditional medicine into evidence-based practice will likely benefit from a focus on mechanistic understanding, standardization of formulations, and identification of bioactive components. Future research should aim to delineate the precise molecular constituents responsible for modulating ICCs pacemaker activity and TRP channel function, assess their efficacy in human IBS patients, and explore synergistic effects with conventional treatments. By bridging traditional knowledge with modern science, herbal formulations such as Rikkunshito may offer novel and effective strategies for treating GI disorders and other multifactorial diseases.

However, there are several limitations to this study. First, while TRP channel modulation was assessed in HEK293T cells, which provide a useful reductionist system to evaluate channel-specific activity by isolating individual TRP channels from endogenous signaling networks, the *in vivo* relevance of these findings requires further validation in native GI and sensory systems, such as ICCs, dorsal root ganglion neurons, or enteric nerve preparations. In this context, the present results should be interpreted as evidence of direct channel-level modulation rather than physiological confirmation in intact tissues. Second, relatively high concentrations of Rikkunshito were required to elicit measurable effects in isolated ICCs preparations, which may not directly reflect *in vivo* exposure levels. Given that Rikkunshito is a complex multi-component extract, the reduced effective concentrations of active constituents in the bath solution, together with the isolated nature of the experimental system, may account for this requirement. Although concentration-dependent effects were consistently observed, potential non-specific membrane actions at higher concentrations cannot be completely excluded. Third, the effects of Rikkunshito on other neuropeptides and inflammatory mediators not measured in this study remain unclear. In addition, although major plant metabolites were quantitatively analyzed using UPLC, no direct correlation analysis between individual metabolites and the observed pharmacological activities was performed. Given the multi-component nature of Rikkunshito, the therapeutic effects are likely to arise from synergistic or complementary interactions among multiple constituents rather than a single active metabolite. Therefore, further studies employing fractionation, bioactivity-guided isolation, or network pharmacology approaches will be necessary to identify key active metabolites and clarify their specific contributions. Finally, the precise molecular components responsible for TRP channel modulation and ICCs regulation have not yet been identified, warranting future studies using isolated compounds or fractionated extracts.

## 5 Conclusion

Our results demonstrate that Rikkunshito alleviates zymosan-induced IBS symptoms through a combination of anti-inflammatory, neuroendocrine, and electrophysiological mechanisms. Specifically, Rikkunshito modulates colonic ICCs pacemaker activity via ghrelin receptor–dependent G protein–PLC–IP_3_–Ca^2+^ signaling, independent of PKC, and selectively regulates TRP channel function. These coordinated actions provide novel insights into the cellular and molecular basis of its therapeutic effects. Collectively, the findings support the potential of Rikkunshito as a multimodal treatment strategy for IBS and lay the groundwork for future mechanistic and translational investigations.

## Data Availability

The raw data supporting the conclusions of this article will be made available by the authors, without undue reservation.
