# Supplementary material for: Rikkunshito ameliorates colonic dysfunction and hormonal imbalance in an IBS mouse model with modulation of transient receptor potential channel activity
Source: Front Pharmacol. 2026 May 22;17:1813619. doi: 10.3389/fphar.2026.1813619 (PMC13237641; doi:10.3389/fphar.2026.1813619)
Supplement: Supplementary file 1 [file Table1.docx]

Supplementary Material

**Table S1. Primer sequences used for qPCR analysis**

| Gene | Forward | Reverse |
| --- | --- | --- |
| mouse *Aqp3* | 5'-ACCCTGCCCGTGACTTTG-3' | 5'-ACACCAGCGATGGAACCC-3' |
| mouse *Aqp4* | 5'-GCATTTCACTCACGGCTCT-3' | 5'-CTCTTGGGAACGGCACTA-3' |
| mouse *Aqp8* | 5'-TGTGTAGTATGGACCTACCTGAG-3' | 5'-ACCGATAGACATCCGATGAAGAT-3' |
| mouse *Zo1* | 5'-CCACCTCTGTCCAGCTCTTC-3' | 5'-CACCGGAGTGATGGTTTTCT-3' |
| mouse *Ocln* | 5'-CCTCCAATGGCAAAGTGAAT-3' | 5'-CTCCCCACCTGTCGTGTAGT-3' |
| mouse *Cldn1* | 5'-TCCTTGCTGAATCTGAACA-3' | 5'-AGCCATCCACATCTTCTG-3' |
| mouse *Gapdh* | 5'-CATGGCCTTCCGTGTTCCT-3' | 5'-CCTGCTTCACCACCTTCTTGA-3' |
